# Pharmacovigilance and tuberculosis: applying the lessons of thioacetazone

**DOI:** 10.2471/BLT.14.142570

**Published:** 2014-10-07

**Authors:** Dennis Falzon, Geraldine Hill, Shanthi N Pal, Wimon Suwankesawong, Ernesto Jaramillo

**Affiliations:** a Global TB Programme, World Health Organization, avenue Appia 20, 1211 Geneva 27, Switzerland.; b Uppsala Monitoring Centre, Uppsala, Sweden.; c Essential Medicines and Health Products, World Health Organization, Geneva, Switzerland.; d National Pharmacovigilance Centre, Bangkok, Thailand.

Following its introduction in the late 1940s, thioacetazone was widely-used as an anti-tuberculosis medicine in the following decades.^1^ Reports of cutaneous hypersensitivity reactions related to its use emerged in the literature soon after its introduction and by the early 1970s the association between the medicine and the adverse drug reaction was well established.^2^ Despite the increased recognition of this risk, thioacetazone remained in use mainly in low-income countries because of its low cost.^3^

In the late 1980s and early 1990s, reports appeared from Africa describing an increased risk of severe cutaneous reactions associated with the use of thioacetazone in persons with human immunodeficiency virus (HIV) infection.^4^^,^^5^ At that time, HIV infection had already reached epidemic proportions in many African countries. Among children and adults with HIV infection and tuberculosis, high fatality was observed in those who developed Stevens-Johnson syndrome or toxic epidermal necrolysis when treated with regimens containing thioacetazone.^4^^,^^5^ In 1991, the World Health Organization (WHO) recommended replacing thioacetazone with ethambutol in patients with known or suspected HIV infection.^6^ Thioacetazone is no longer included in WHO’s recommended first line treatment for tuberculosis and is now reserved for uncommon situations in which treatment options have been compromised by resistance to other anti-tuberculosis medicines in HIV-negative individuals.^7^

For more than 50 years, WHO has been encouraging countries to reinforce their pharmacovigilance – the surveillance of adverse drug reactions – at national level.^8^ Nonetheless, under-reporting is a major problem, both in developed and developing countries. Computing adverse drug reaction frequencies for a particular medicine is impossible with spontaneous reporting, as there is no way to obtain a denominator – an accurate estimate of the number of people exposed to the drug. Moreover, incomplete reports mean that it is difficult to establish a relationship between the suspected medicine and the adverse drug reaction.

Currently the WHO Programme for International Drug Monitoring – a worldwide pharmacovigilance network – receives individual case safety reports from the national drug safety authorities of 118 countries. Spontaneous reports of adverse drug reactions, submitted by participating countries are gathered in the database VigiBase™, which is maintained by the WHO Collaborating Centre, the Uppsala Monitoring Centre in Sweden.^9^ VigiBase™ has accumulated well over nine million individual case safety reports since the late 1960s, over half of which have been received in the last five years. This reflects an improved regulatory environment, greater awareness of the need for drug safety monitoring, and the expansion of the WHO Programme for International Drug Monitoring. VigiBase™ currently accrues about 800 000 individual case safety reports each year.^10^

**Fig. 1:**
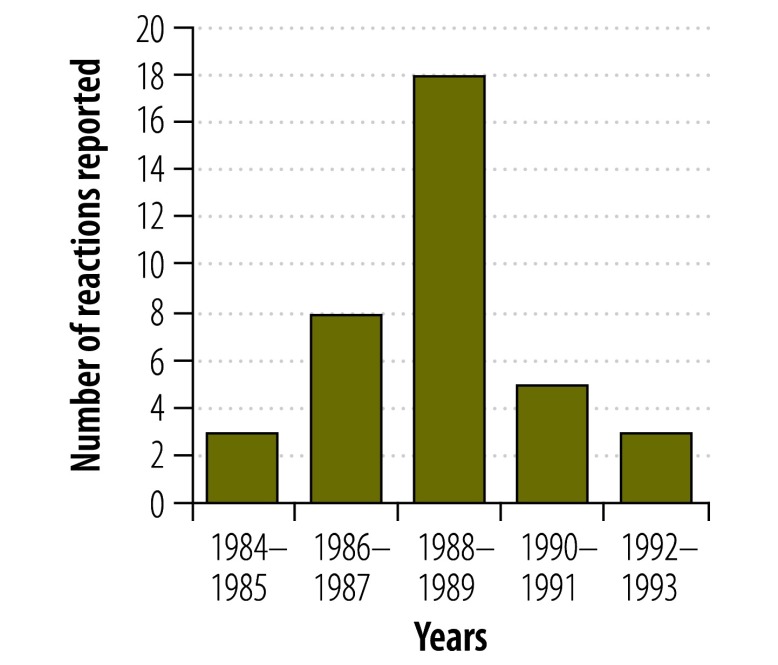
Reported cutaneous hypersensitivity reactions^a^ associated with thioacetazone, Thailand, 1984–1993

^a ^Bullous eruption (*n* = 1); Erythema multiforme (*n* = 2); Exfoliative dermatitis (*n* = 14); Stevens-Johnson syndrome (*n* = 19); Toxic epidermal necrolysis (*n* = 1).

We searched VigiBase^™^ for adverse drug reactions related to thioacetazone and found an increase in reports of skin conditions, some of them reported as severe, in Thailand during the 1980s and early 1990s (Fig. 1). These reports are consistent with – and even predate – the publications from Africa. While there is no information about whether these conditions were occurring in people with HIV infection, it is notable that the timing of the reporting peak coincides with the evolution of the HIV epidemic in Thailand.^11^ Isolated reports of cutaneous hypersensitivity were reported by other countries during this time but no other country reported as large an increase as Thailand.

Data source: VigiBase^™^^9^

The lessons learnt from the thioacetazone episode remain pertinent today; medicines that have been in use for a long time need to be monitored for safety when used for new indications or in a population with different co-morbidity profiles. The spontaneous reports from Thailand show the usefulness of databases for pharmacovigilance, despite the limitations of these reports.

The treatment of drug-resistant tuberculosis is now poised to undergo changes. More patients in more countries will be receiving treatment in the coming years and important modifications in the regimen composition and duration will be increasingly introduced. Current treatment regimens for these patients are long and complex and their toxicity when used in certain patient subgroups may not be completely profiled.^7^^,^^12^ Several of these patients will also be treated for other co-morbidities, including HIV infection, at the same time. The introduction of new drugs and the repurposing of medicines beyond their primary indication are expected to become more widespread as tuberculosis programmes strive to improve outcomes for their patients. Two new medicines – bedaquiline and delamanid – have recently been approved by the United States’ Food and Drug Administration and/or the European Medicines Agency, for use in multidrug-resistant tuberculosis patients.^13^^,^^14^ Other drugs that have not yet completed phase 3 clinical trials for tuberculosis are already being included in treatment regimens. In addition, more medicines are expected to be released shortly.^12^ The clinical use of these new medicines will likely generate adverse reactions or drug interactions that are yet unknown.

Until now, pharmacovigilance has had a low priority among the activities of national tuberculosis programmes and it is not yet an integral part of the framework used to assess tuberculosis programme performance.^15^ The developments in tuberculosis treatment are compelling reasons to integrate pharmacovigilance with other routine monitoring and operational research components of tuberculosis programmes. The different pharmacovigilance methods that can be used in such programmes have been detailed by WHO.^16^^,^^17^ However, these methods do not detract from the importance of practitioners reporting potential harms in the medical literature.

Active surveillance techniques such as Cohort Event Monitoring are now recommended by WHO to use when new drugs and regimens are introduced.^7^ Instructions on how to operationalize pharmacovigilance in tuberculosis programmes are being worked out with countries and technical partners to ensure that adverse drug reactions are detected faster and managed early. Integration of pharmacovigilance in the programmes will require an effective collaboration between the tuberculosis control programme and the drug safety authority at country level. It will also require active participation in the WHO Programme for International Drug Monitoring. Investment in technologies that enable the proper monitoring of patients, including laboratory diagnostics and the collection and analysis of data will be necessary. Health-care workers need to acquire new skills to undertake sound pharmacovigilance.

Among the leading donors supporting tuberculosis control efforts, the Global Fund to Fight AIDS, Tuberculosis and Malaria has been a long-standing proponent for strengthening pharmacovigilance within treatment programmes.^18^ Nonetheless, actual investment by national tuberculosis programmes in pharmacovigilance has fallen short.^19^ It is therefore important that in any funding proposal for novel or repurposed tuberculosis medicines and/or for the introduction of new tuberculosis regimens, tuberculosis programmes and technical partners incorporate activities that reinforce pharmacovigilance. These activities need to be realistically budgeted for to be able to increase patient safety and to avoid future incidents like the thioacetazone episode.
